# Advanced Lung Adenocarcinoma Patient with *ERBB2* Amplification Identified by Comprehensive Genomic Profiling Benefits from Trastuzumab

**DOI:** 10.1155/2020/9072173

**Published:** 2020-03-01

**Authors:** Chi-Wei Tao, Mei-Yin Chen, Ching-Min Tseng, Nina Lapke, Shu-Jen Chen, Kien Thiam Tan

**Affiliations:** ^1^Division of Respiratory Therapy, Department of Internal Medicine, Cheng-Hsin General Hospital, Taipei, Taiwan; ^2^ACT Genomics, Co. Ltd., Taipei, Taiwan

## Abstract

For non-small-cell lung cancer (NSCLC) patients without established actionable alterations in genes such as *EGFR* or *ALK*, options for targeted therapy remain limited in clinical practice. About 5% of lung adenocarcinoma patients have tumors with *ERBB2* genetic alterations, with even fewer patients harboring *ERBB2* amplification. Currently, clinical trials mainly use IHC, FISH, or mutation testing to identify potential responders to ERBB2-targeting agents. The use of next-generation sequencing (NGS) to detect *ERBB2* alterations, including copy number variants, is rare. In this study, we present an EGFR- and ALK-negative advanced NSCLC case for which we conducted comprehensive tumor genomic profiling to identify potentially actionable alterations. The tumor harbored an *ERBB2* amplification, and trastuzumab-based therapy resulted in an excellent response, with a necrotic regression of the patient's lung lesion. Although he developed brain metastasis four months after trastuzumab initiation, he survived for an additional period of eight months without local recurrence or other systemic metastasis. This case report shows that the use of comprehensive genetic testing enables the identification of rare actionable alterations in NSCLC patients without other options for targeted treatment.

## 1. Introduction

Non-small-cell lung cancer (NSCLC) patients who do not have tumor genetic alterations sensitizing them to established targeted therapies, e.g., alterations of *EGFR* or *ALK*, often do not receive targeted therapy. Although driver alterations, such as *ERBB2* alterations, may exist in some of those patients, those are usually not used to guide therapy in clinical practice. Furthermore, it is not entirely clear which markers are best to identify responders.

*ERBB2* genetic alterations could be drivers in about 5% of lung adenocarcinoma [1, 2] and can be divided into *ERBB2* mutations and *ERBB2* amplification, with rare or no overlap between the two [1–3]. NCCN guidelines list trastuzumab as an emerging targeted agent for *ERBB2* mutations; however, amplifications are not currently included as actionable alterations. There is no consensus about the best method to detect ERBB2-driven tumors. Targeted therapy response rates are difficult to determine due to low patient numbers but differ with used therapeutic agents and markers [4–10]. Used markers include IHC for protein expression, mass spectrometry or next-generation sequencing (NGS) for mutation detection, and FISH or NGS for amplification detection. Recent studies found trastuzumab emtansine response rates of about 33% for *ERBB2* mutant and 20% for FISH-positive patients [4, 5]. NGS has rarely been used for *ERBB2* amplification detection in clinical studies, although a study showed that two out of three patients with an NGS-detected *ERBB2* amplification responded to therapy [4]. However, NGS has a good performance when compared to other methods of amplification detection [2, 11]. A further advantage of using NGS to identify therapeutic options for individual patients outside of clinical trials is that NGS can detect different types of genetic alterations while including many genes in a single test. This is particularly important when the frequency of alterations for individual driver genes is relatively low.

Here, we report a case of advanced EGFR- and ALK-negative NSCLC for which comprehensive tumor genomic profiling identified that an *ERBB2* amplification and treatment with a trastuzumab-based regimen resulted in an excellent outcome. Our study demonstrates the value of broad genetic testing to detect actionable genetic alterations present in NSCLC patients who are ineligible for targeted therapies after standard testing.

## 2. Case Presentation

**Figure 1:**
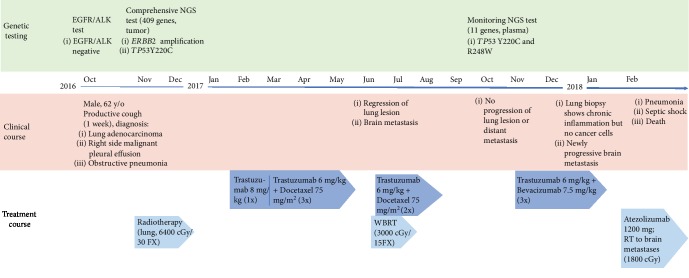
Clinical time course for a non-small-cell lung cancer patient treated with trastuzumab. The time course for disease and treatment and the results of performed genetic testing are shown starting from the time of diagnosis. Abbreviations: NGS: next-generation sequencing; WBRT: whole brain radiotherapy.

In October 2016, a male, 62-year-old nonsmoker and nondrinker presented with productive cough that had lasted for a week. He was diagnosed with stage IVA right upper lobe lung adenocarcinoma (cT4N2M1a, ECOG 0), with obstructive pneumonia and right-side malignant pleural effusion. The time course of his disease starting from diagnosis and his treatment is displayed in Figure 1. The patient's lesion was found to be EGFR wild-type and ALK-negative by standard clinical testing. Furthermore, chemotherapy was not considered due to the patient's pneumonia, which was treated by antibiotics. Instead, the patient initially underwent radiotherapy (6400 cGy/30 FX).

In agreement with current guidelines, broad molecular profiling was performed to identify treatment-relevant genomic alterations, and informed consent was obtained for the use of the resulting data. For analysis, a formalin-fixed paraffin-embedded (FFPE) sample biopsied from the right upper lobe was used. Areas with high tumor content were identified by H&E stain, and subsequently, a macrodissection was performed to enhance the tumor cell proportion. The ACTOnco™ panel from ACT Genomics, Ltd. was used for comprehensive genetic testing. The assay performs next-generation sequencing of all coding exons of 409 cancer-related genes to detect single nucleotide variants, small insertions and deletions, and copy number variants. Details regarding this panel have been previously published [12]. Sequence variants with a coverage of at least 25 reads and an allele frequency of ≥5% for regular variants and ≥2% for actionable variants were considered. An additional NGS test able to detect the presence of 72 known fusion transcripts for *ALK*, *ROS1*, *RET*, and *NTRK* fusion genes was also performed.

**Figure 2:**
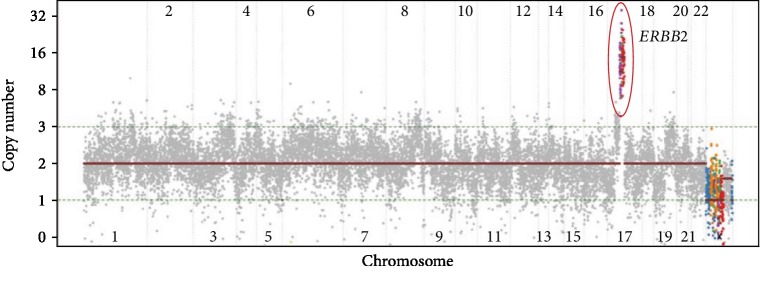
Copy number profile of non-small-cell lung cancer tumor sample identifies *ERBB2* amplification as a potentially actionable alteration. A tumor sample from the patient's lung was used to perform comprehensive genomic profiling. The resulting copy number profile is shown, with observed copy numbers being displayed on the *y*-axis. The red circle indicates the amplification of a genomic region that includes the *ERBB2* gene.

There were no fusion genes detected in the patient's sample. However, 27 sequence variants, including *TP53* Y220C, were identified (Table 1). The tumor had a stable copy number profile, and no copy number gains or losses were detected, with the exception of amplification of cytoband 17q12. This amplification included *CDK12*, *PGAP3*, and the clinically relevant gene *ERBB2* (Figure 2). The observed copy numbers for those genes were 11.5, 15, and 15, respectively. However, the observed copy number does not take tumor purity into account, and a copy number of 45 copies for *ERBB2* were calculated based on the estimated tumor purity of 30%. These results suggested an *ERBB2*-driven tumor and a potential benefit from therapies targeting ERBB2, such as trastuzumab.

**Figure 3:**
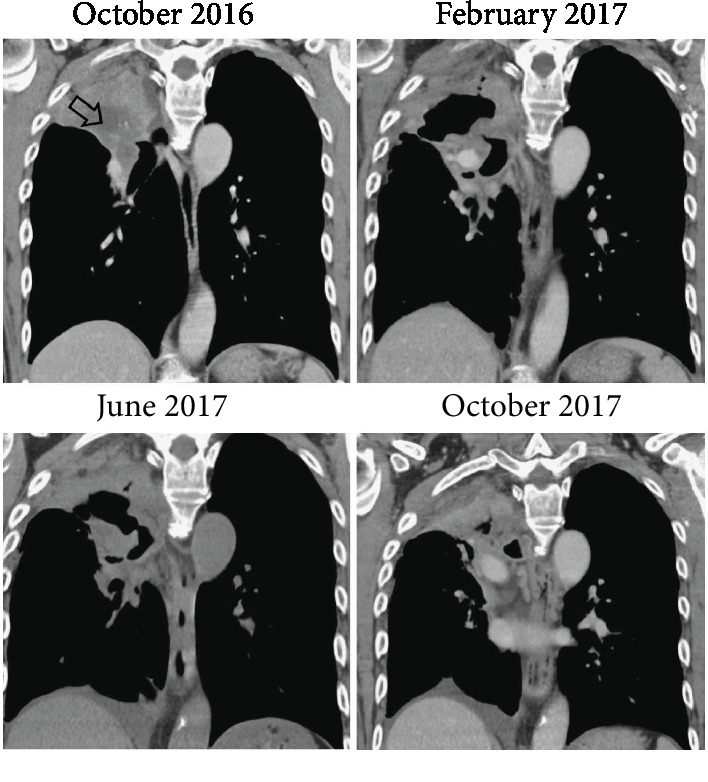
Lung imaging results during the course of the disease. Chest CT imaging is shown for the time between October 2016 and October 2017. For the October 2016 CT image, the lesion is indicated by an arrow.

The patient started trastuzumab treatment in February 2017. Trastuzumab was first administered once as monotherapy without concurrent chemotherapy due to the patient's obstructive pneumonia. After the patient's pneumonia improved, the patient received three more doses of trastuzumab with concurrent docetaxel treatment. CT imaging in June 2017 demonstrated a necrotic regression of the right upper lobe lung lesion after the initiation of trastuzumab treatment (Figure 3). The restaging in July 2017 confirmed the absence of local recurrence. However, the patient had developed brain metastasis. There was no evidence for other systemic metastasis. Since trastuzumab and docetaxel do not effectively pass through the blood-brain barrier, whole brain radiotherapy (WBRT; 3000 cGy/15FX) was initiated in addition to the continuation of trastuzumab and docetaxel. In October 2017, a follow-up was performed. At that time, the patient's ECOG performance status was 0. CT imaging of the lung showed no evidence of local progression or distant metastasis. However, the patient's blood was used for the ACTMonitor™ Lung 11 gene NGS circulating tumor DNA (ctDNA) test. This test covers hotspots in the genes *ALK*, *BRAF*, *CDKN2A*, *CTNNB1*, *EGFR*, *ERBB2*, *KRAS*, *MET*, *PIK3CA*, *TP53*, and *U2AF1*. The results showed the *TP53* Y220C mutation that had previously been detected in the FFPE tumor specimen and an additional *TP53* R248W mutation at allelic frequencies of 4.4% and 1.1%, respectively. The detection of tumor-specific variants in plasma upon therapy is associated with less favorable treatment outcomes [13], and our results indicated the possibility of a future disease progression. The patient was treated with combined bevacizumab and trastuzumab therapy, and the treatment was given three times until January 2018. At that time, CT imaging of the brain indicated a newly progressive brain metastasis. A bronchoscopic exam and serial staging studies did not indicate local tumor recurrence or other metastasis, and the obtained specimen from a bronchoscopic biopsy showed chronic inflammation but no presence of cancer cells. The patient was treated with RT for the brain metastases and atezolizumab in early February 2018. However, he developed pneumonia, followed by septic shock, and died in mid-February.

## 3. Discussion

In this case report, we identified *ERBB2* amplification in an EGFR- and ALK-negative advanced NSCLC patient. The patient's lung lesion responded well to trastuzumab-based treatment, and there was no evidence of cancer cells in a lung biopsy 12 months after trastuzumab treatment initiation at the time of the patient's death.

The results of the genetic testing suggested an ERBB2-driven tumor and furthermore revealed the presence of the *TP53* Y220C variant. *TP53* mutations could have implications for cancer treatment, although their actionability is less well studied than that of *ERBB2* amplification. *TP53* mutation may be associated with an increased benefit from bevacizumab [14]. Furthermore, a decreased benefit from platinum was suggested; however, the effect might depend on additional factors, such as histology and mutation types [15, 16]. *In vitro* data show an association between the variant *TP53* Y220C and reduced sensitivity to platinum, an effect that was not observed for taxane [17].

Our patient benefitted from trastuzumab-based treatment, but radiation and docetaxel may have contributed to the encouraging treatment outcome. However, docetaxel response rates were reported to be moderate in NSCLC [18, 19]. Furthermore, radiotherapy outcomes have been less encouraging in patients with *TP53* mutations [20], and radiation to our patient's brain metastasis had limited effectivity. Therefore, it is likely that our patient's treatment response in the lung was mainly driven by trastuzumab, or that trastuzumab provided an added treatment benefit. Although brain disease progressed during the treatment, our patient survived for eight months after the diagnosis of brain metastasis, which is a relatively long survival time [21]. The improved survival is probably due to systemic effects of trastuzumab, although some reports indicate that when used in combination with WBRT, brain metastases may be sensitized to trastuzumab treatment [22, 23]. However, small molecule tyrosine kinase inhibitors such as afatinib, an EGFR- and ERBB2-targeting agent, may be a valuable alternative for targeted treatment of patients with brain metastasis [24]. It would also have been interesting to evaluate whether the brain lesions differed from the lung lesion in the genetic actionability profile. In this regard, genetic testing of cerebrospinal fluid could have been considered [25].

In conclusion, our case report shows that NGS could successfully identify *ERBB2* amplification as an actionable rare driver alteration for an advanced NSCLC patient. The patient survived for a total of 16 months. He responded to trastuzumab-based therapy initiated after four months and remained without apparent lung lesions until death. Future studies using NGS to clinically guide treatment of NSCLC patients with actionable alterations such as *ERBB2* amplification are eagerly awaited.

## Figures and Tables

**Table 1:** List of sequence variants detected in the patient's tumor sample.

Gene	Variant
*TP53*	Y220C
*NUMA1*	E1151K
*G6PD*	H32R
*ERBB2*	R456L
*PMS2*	Q643R
*RNF213*	E4988Q
*RNF213*	I3234R
*TRRAP*	E2732D
*KAT6A*	R507C
*PTCH1*	S827G
*MCL1*	P65L
*MAGI1*	A614V
*NOTCH4*	R937S
*FGFR3*	A429T
*TRRAP*	I1141V
*PBRM1*	K925I
*CDH1*	P88R
*KMT2C*	A3921V
*CBL*	G14C
*LRP1B*	S1251I
*SYNE1*	N8455S
*LRP1B*	Splice acceptor
*IDH2*	G383R
*TAL1*	D78N
*LPHN3*	E1324Q
*EXT1*	P477L
*TRRAP*	D428N
